# Sensor Topology-Aware Three-Branch Fusion for sEMG Gesture Recognition

**DOI:** 10.3390/s26154733

**Published:** 2026-07-26

**Authors:** Luoqi Cui, Yong Liu, Hadi Fathollahi Abdar, Xinqin Gao, Mingshun Yang, Mohammad Reza Chalak Qazani

**Affiliations:** 1 School of Mechanical Engineering, Xi’an University of Technology, Xi’an 710048, China or luoqi.cui@my.jcu.edu.au (L.C.); gaoxinqin@xaut.edu.cn (X.G.); yangmingshun@xaut.edu.cn (M.Y.); 2 College of Science and Engineering, James Cook University, Townsville, QLD 4815, Australia; 3 Faculty of Computing and Information Technology (FCIT), Sohar University, Sohar 311, Oman; h.fathollahi.abdar@gmail.com

**Keywords:** surface electromyography (sEMG), hand gesture recognition, multi-branch fusion network, adaptive feature fusion, reliability-aware fusion, deep learning

## Abstract

Surface electromyography (sEMG) is increasingly used for gesture recognition in prosthetics, rehabilitation, and human–computer interaction. Existing architectures typically force heterogeneous sEMG features into a shared latent representation, limiting their ability to capture complementary temporal, frequency-domain, and inter-electrode spatial dependencies. To better exploit these features, this paper proposes a three-branch fusion network. Unlike many existing multi-branch methods, the proposed network explicitly models the ring arrangement of armband electrodes, capturing the adjacency information in the sensor topology that linear channel representations ignore. The temporal and spectral branches use a compact multi-scale residual structure, so this topology branch is added while maintaining modest model complexity. A reliability-aware routing mechanism then adaptively assigns fusion weights to the three branches for each sample. On NinaPro DB5 Exercise B (eight-channel lower armband), the method reaches 83.99% under subject-dependent training and 85.66% under transfer learning, exceeding prior transfer learning approaches under matched conditions. Ablation experiments confirm that the three branches contribute non-redundant information and that adaptive fusion outperforms fixed combinations. The architecture also generalizes to MyoArmbandDataset under a subject-adaptive transfer learning protocol without dataset-specific hyperparameter retuning, indicating potential for wearable gesture interfaces, rehabilitation, and prosthetic control.

## 1. Introduction

Surface electromyography (sEMG) signals capture electrical activity generated by muscle contractions using non-invasive electrodes placed on the skin. Due to its non-invasive nature, intuitive representation of movement intentions, and increasing availability through low-cost wearable devices, sEMG has become an important sensing modality for hand gesture recognition and natural human–computer interaction [1,2]. Its applications span prosthetic control, rehabilitation, human–computer interaction, and emerging remote-control scenarios such as drone operation [3,4,5]. Among them, forearm sEMG gesture recognition using armband electrodes has attracted widespread attention because it provides a convenient, minimally invasive way to translate muscle intentions into device commands [6].

sEMG signals contain complementary information from the temporal, spectral, and spatial dimensions. In the temporal dimension, the waveform within a recording window reflects the discharge behavior of motor units and the resulting muscle activation dynamics [7]. In the spectral dimension, different muscle activation states may show different energy distributions across frequency bands. Classical feature processing methods, such as mean frequency, root mean square (RMS), and wavelet-based features, have therefore been used to describe these frequency-related patterns [8]. In the spatial dimension, non-invasive electrodes are placed around the forearm, partially overlapping muscle groups [9]. The relative activation patterns across the electrode ring can provide discriminative information about muscle involvement in different gestures [10,11]. These three dimensions provide distinct but complementary information. Individual finger flexions that produce similar overall activation envelopes in the temporal domain can often be separated by their spectral characteristics, and gestures with comparable spectral content may still differ in their spatial activation patterns across the electrode ring.

Despite the multi-dimensional nature of sEMG signals, many deep learning-based sEMG recognition algorithms still rely on a single representation path. Early convolutional neural network (CNN)-based methods treated raw multi-channel sEMG signals as image-like inputs, allowing the network to learn features end-to-end [10,12]. However, their cross-subject generalizability remained limited. Transfer learning methods later improved cross-subject generalization, but most of them still used a single input path, such as raw signals, spectrograms, or continuous wavelet transform (CWT) representations [12,13]. Time–frequency methods converted sEMG signals into CWT scalograms or spectrograms and then applied two-dimensional (2D) CNNs to the transformed inputs [12]. More recent studies have also adopted sequence models or Transformer-based architectures on time-domain channel representations [14]. These methods have improved the recognition accuracy and, in some cases, introduced frequency-domain information. However, they still often require the model to learn temporal, spectral, and spatial cues from a single input stream or a single transformed view. This increases the burden on representation learning, especially when subject-specific training data are limited. The limitation becomes more evident in complex multi-gesture recognition tasks. For example, studies using Myo-based public datasets with seven gesture classes often report high recognition accuracy. In contrast, the performance usually drops when related models are extended to NinaPro DB5 protocols with a larger gesture set, such as 18 classes [6,12,15].

A natural alternative is to assign a dedicated pathway to each representation type and allow the network to learn how to integrate them. Parallel multi-branch architectures extract features from different signal views and fuse their outputs. This idea has been explored in related areas, including skeleton-based action recognition and biomedical signal classification [16,17]. In sEMG gesture recognition, recent studies have also proposed multi-stream or multi-branch models. Examples include multi-dimensional branch fusion networks, adaptive multi-stream CNNs, and multi-view transformed-image multi-stream CNNs [18,19,20]. However, this direction has not yet been systematically examined for the explicit separation of temporal, spectral, and topological representations. It also remains unclear how different fusion mechanisms affect performance when the same branch set is used. Several representative sEMG fusion designs still use preset operations, such as concatenation or decision-level fusion [21,22]. Therefore, this study investigates whether temporal, spectral, and topological representations provide non-redundant information for sEMG gesture recognition. It further examines how the outputs of multiple complementary branches should be combined and whether the fusion mechanism affects performance beyond simply increasing the number of branches.

To address these issues, this paper proposes the Ring Topology and Reliability Fusion Network (RTF-Net) for sEMG gesture recognition. Multi-branch fusion is by now well studied, and temporal and spectral representations are firmly established as effective. The ring geometry of the armband has not gone unnoticed: circular padding has been used to preserve first-to-last electrode adjacency within a single convolutional stream [13]. However, the topology enters as a layer-level implementation choice rather than as a representation branch whose independent contribution is measured. What has not been examined is whether the closed-ring topology warrants a dedicated pathway alongside temporal and spectral views and whether the three then carry non-redundant information. RTF-Net retains the two established representations and adds the missing piece. A temporal branch captures motor-unit firing dynamics through multi-scale residual convolution, a spectral branch extracts frequency-band components through a learnable filter bank, and a topology branch preserves the circular electrode neighborhood through circular convolution. The branches are fused through a learnable module that adaptively weights each representation by its reliability.

The individual components, including dilated residual convolution, learnable filter banks, and softmax-gated fusion, are established techniques. The contribution of this work lies in how they are arranged for armband-based sEMG and in the evidence that the resulting branches carry non-redundant information. The main contributions of this paper are as follows.

(1) A sensor topology-aware representation branch: This branch encodes the spatial dependencies among electrodes that arise from the ring layout of the armband. Through circular convolution applied at every layer, it preserves the wrap-around adjacency between the first and last electrodes, yielding a spatial representation that is complementary to the temporal and spectral branches.
(2) A sample-adaptive fusion mechanism guided by branch-level reliability cues: The router uses branch-level confidence and predictive entropy to adjust the relative contribution of each branch for each input sample, rather than relying on a single set of fixed global weights.
(3) A compact three-branch implementation: A multi-scale residual structure is adopted in the temporal and spectral branches to control parameter growth. The added topology branch and routing are therefore incorporated without a significant increase in model complexity, keeping the network lightweight.

The remainder of this paper is organized as follows: Section 2 reviews relevant work on sEMG gesture recognition. Section 3 describes the dataset used in this study. Section 4 details the proposed method. Section 5 presents the experimental results, including ablation experiments and generalization validation. Section 6 summarizes the entire paper and discusses future research directions.

## 2. Related Work

Atzori et al. [23] introduced an early CNN baseline for movement classification on the NinaPro database. Their study demonstrated that short multi-channel sEMG windows can be processed by CNNs for end-to-end feature learning, thereby reducing the dependence on manually designed descriptors. However, the CNN architecture was relatively shallow and was not specifically designed to capture long-range temporal dependencies. To improve subject-level generalization, Côté-Allard et al. [12] proposed a cross-subject transfer learning framework for sEMG hand gesture recognition. They showed that pre-training on a pool of source subjects can reduce the calibration data required for a new subject, although each input window was still processed through a single representation pathway. Subsequent studies further improved single-path feature extraction; for example, Chen et al. [24] proposed a compact CNN for sEMG gesture recognition. Temporal modeling was then explored through recurrent and hybrid architectures, such as long short-term memory (LSTM)–CNN models [25]. More recently, Transformer-based models and attention mechanisms have been introduced to model temporal-channel dependencies in sEMG or high-density sEMG signals [26,27]. In parallel, Celik and Bayazit [15] focused on the training and adaptation strategy, combining transfer learning with similarity learning to improve gesture recognition with limited subject-specific data. Although these methods improve performance from different perspectives, including temporal modeling, transfer learning, attention-based feature extraction, and calibration-efficient training, they largely retain a common architectural premise: temporal, spectral, and spatial cues enter the network through a single input pathway and must be disentangled implicitly by learned features.

Another research direction attempts to reduce the burden of implicit feature discovery by representing sEMG signals through explicit time–frequency transformations. Côté-Allard et al. [12] evaluated raw sEMG, spectrogram, and CWT inputs within a transfer learning framework. Chen et al. [24] further used CWT-based spectral inputs with a compact CNN architecture, making spectral information more explicit than in raw-signal CNNs. However, these methods still generally allow only one representation view to enter the network at a time. To overcome this single-view limitation, Shin et al. [28] proposed a multi-stream sEMG architecture in which time-varying and frame-wise features are processed in parallel before fusion. This finding supports the value of combining complementary feature views. Nevertheless, existing sEMG multi-stream methods still tend to focus on a limited set of representations and often rely on fixed fusion operations, such as concatenation, averaging, or predefined feature aggregation [21]. Therefore, two questions remain unresolved: which representation should complement the established temporal and spectral views and how these branches should be combined when their reliability varies across gestures and subjects.

Recent sEMG multi-branch designs differ mainly in which views they separate and how they recombine them. SGRN [18] pairs a spatial network with a temporal network and fuses them in a dedicated spatiotemporal module, without a spectral view. MSACNN-RM [19] combines multiple convolutional streams with adaptive convolution kernels and residual blocks; its adaptivity operates within the streams rather than on their combination. Zhang et al. [20] encode the same signal as Sigimg, GADF, and MTF images and merge the streams with a fixed strategy. Beyond these, MFN-SPSCA [29] models the armband channel structure by graph convolution under a spatial partitioning strategy and weights time–space–frequency features by cross-attention but fuses two sensing modalities (sEMG and accelerometer), so that branch complementarity follows in part from the sensors themselves; the present work uses sEMG alone and no inertial data. Fratti et al. [13] retain the ring geometry through circular padding, but within a single multi-scale stream. Across these designs, the electrode ring is either absorbed into a general spatial graph or handled as padding, and recombination is either fixed or based on feature-level attention rather than on per-sample gating conditioned on branch reliability.

The problem of fusing parallel feature streams has been widely studied in multimodal and multi-view learning, with strategies ranging from simple concatenation and averaging to learned weighting and attention-based fusion. Conditional computation methods, such as the sparsely gated mixture-of-experts layer, further allow the fusion behavior to depend on each input sample, routing samples to different experts according to a learned gating function [30]. In sEMG-related fusion studies, however, fusion is often designed for multimodal sensor combinations, such as sEMG and accelerometer signals [31], or implemented using fixed or weakly adaptive feature combination rules, such as concatenating parallel feature streams [21]. Therefore, although adaptive sample-wise fusion has been extensively studied in the broader machine learning literature, its role in armband-based sEMG gesture recognition has not been systematically examined. It is still unclear whether input-conditional fusion provides additional benefits over fixed fusion when the parallel branches represent different intrinsic views of the same sEMG signal.

A limited number of studies have explicitly considered the spatial structure of multi-channel sEMG signals. For high-density sEMG acquired with two-dimensional electrode arrays, the natural electrode arrangement can be mapped to two-dimensional images, allowing the use of standard two-dimensional convolutional kernels or Vision Transformer architectures to preserve part of the spatial geometry [27,32]. However, armband sensors have a different spatial structure. Their electrodes are arranged in a loop around the forearm, meaning that the channel relationship is cyclic rather than purely linear or planar. Few studies have explicitly encoded this cyclic topology in convolutional layers for armband-based sEMG gesture recognition. Existing approaches usually process channels as a linear axis, rely on global pooling or fully connected layers for cross-channel interaction, or inherit spatial processing assumptions from high-density sEMG designs. This leaves the circular topology of armband electrodes underutilized.

Circular convolution along the electrode axis is a principled choice rather than an ad hoc design. The operation is therefore equivariant to armband rotations around the forearm. Group-equivariant CNNs [33] and the cyclic symmetry operations in [34] extend this idea to broader symmetry groups. A natural extension for the electrode ring is the dihedral group, which would also model reflection caused by reversed placement or use on the contralateral arm. This extension remains to be evaluated. Toroidal convolution is not suitable because only the electrode axis is periodic, whereas the temporal axis is not.

## 3. Datasets

This study evaluated the proposed method using two publicly available sEMG datasets: NinaPro DB5 Exercise B [6] as the primary benchmark dataset and MyoArmbandDataset [12] as a secondary armband dataset for generalization evaluation. Both datasets were acquired using consumer-grade Myo armbands with dry stainless-steel electrodes, providing eight sEMG channels per armband at a sampling rate of 200 Hz. The two datasets therefore share the same hardware platform, maintaining consistency in the underlying sensing approach while isolating the influence of dataset-specific factors such as recording protocols, gesture vocabulary, and subject composition. The two public datasets are used independently with different evaluation protocols based on their own designs. The primary NinaPro DB5 protocol adopted in this study is subject-dependent, meaning that each subject’s model is trained from scratch using that subject’s own data. MyoArmbandDataset, on the other hand, follows a subject-adaptive transfer learning protocol. The source model is pre-trained on another set of subjects’ data and then calibrated and tested using each target subject’s own data. All hyperparameters are fixed on NinaPro DB5 and directly applied to MyoArmbandDataset without readjustment. See Section 3.1 and Section 3.2 for details.

### 3.1. NinaPro DB5 Exercise B

The NinaPro DB5 dataset [6] is part of the publicly available NinaPro database [35], which provides large-scale sEMG recordings for gesture and motion recognition research. DB5 was collected from 10 healthy subjects using two Myo armbands worn on the same forearm. In the original acquisition setup, one armband was positioned around the forearm near the radio-humeral joint. In contrast, the second armband was placed next to it and rotated by 22.5° to fill the gaps between electrodes. The dataset contains three exercise modules: Exercise A, consisting of 12 basic finger movements; Exercise B, consisting of 8 isometric and isotonic hand configurations and 9 basic wrist movements; and Exercise C, consisting of 23 grasping and functional movements. Together with the rest class, the complete dataset contains 53 labels. In this study, Exercise B was used, which combines 8 hand configurations, 9 wrist movements, and the rest class to form an 18-class classification problem.

Each subject repeated each gesture six times, with each repetition lasting approximately 5 s, followed by a 3 s rest period. This study adopts a subject-dependent NinaPro DB5 Exercise B protocol with the standard repetition split used in previous studies [6,35]. For each subject, repetitions 1–4 were used for training, while repetitions 5–6 were held out for testing. Within the training repetitions, 10% of the windows were randomly held out as a validation set for hyperparameter selection, and the remaining 90% were used for training. The test repetitions were used only for final evaluation and never for tuning or model selection, and the hyperparameter configuration selected here was reused without modification on MyoArmbandDataset (Section 3.2). A separate model was trained from scratch for each subject using only that subject’s training data. No data from the remaining subjects were used during training. Inertial measurement unit (IMU) data and data from the second armband were not included. This setting provides a reproducible benchmark for evaluating recognition performance with limited subject-specific calibration data, while isolating the proposed architecture’s contribution from cross-subject knowledge transfer.

Two electrode configurations were considered. The 8-channel configuration used only the first Myo armband channels, corresponding to the armband placed around the forearm near the radio-humeral joint. This configuration is consistent with the convention adopted in previous DB5-based armband benchmarks [12,15] and serves as the primary experimental setting in this paper. The 16-channel configuration used both Myo armbands simultaneously as a secondary setting to evaluate the effect of increased electrode coverage. In the 16-channel configuration, the two armbands were treated as two independent eight-electrode rings. Circular encoding was applied separately within each physical ring, after which the resulting representations were merged for subsequent processing. The 18-class label space and the training/test partitioning are identical for both configurations.

### 3.2. MyoArmbandDataset

MyoArmbandDataset [12] is a publicly available sEMG dataset designed to study transfer learning across subjects and sessions. The dataset was collected using a single Myo armband worn on the forearm, providing eight dry-electrode sEMG channels at 200 Hz. In the original publication, the dataset comprises two sub-datasets: a pre-training set with 19 able-bodied participants and an evaluation set with 17. Participants performed seven hand and wrist gestures: neutral, radial deviation, wrist flexion, ulnar deviation, wrist extension, hand close, and hand open. All gestures were recorded as static postures.

In this study, MyoArmbandDataset was used as an auxiliary benchmark to evaluate whether the proposed architecture generalizes beyond NinaPro DB5. The study followed the dataset organization used in the original transfer learning protocol [12], using the pre-training subset for source-domain training, training0 for subject-specific calibration, and the union of test0 and test1 for evaluation. This design allows the model to be tested on a second Myo-based armband dataset with a different gesture vocabulary, subject composition, and recording protocol.

## 4. Method

**Figure 1:**
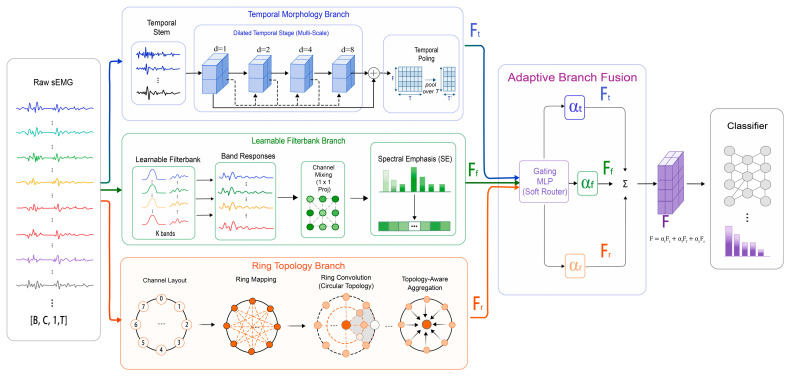
Overall architecture of RTF-Net.

The proposed network maps windowed multi-channel sEMG signals to probability distributions over gesture categories via three parallel branches with distinct inductive biases, followed by an adaptive fusion module and a classifier. The temporal morphology branch, the learnable filter bank branch, and the ring topology branch extract complementary representations of the same input: a multi-scale temporal view, a data-driven spectral decomposition, and a topology-aware spatial view of the electrode ring. Their outputs are weighted by a soft router and combined into a unified representation. The overall architecture is shown in Figure 1.

Let *x* ∈ R^(*B*×*C*×1×*T*)^ denote a preprocessed input window, where *B* is the batch size, *C* = 8 the number of electrode channels, and *T* = 52 the number of temporal samples (260 ms at 200 Hz). The three branches independently transform *x* into feature vectors *F*_t_, *F*_f_, *F*_r_ ∈ R*^D^* with *D* = 128, which are weighted by per-sample gating coefficients *α*_t_, *α*_f_, *α*_r_ and combined into a fused representation *F* that is mapped to the 18 gesture classes by a fully connected classifier. All three branches output 128-dimensional feature vectors. Using the same feature dimensionality ensures that the temporal, filter bank, and ring topology representations are directly compatible with the weighted fusion module and keeps the branch interfaces consistent across all experiments. The feature dimension D = 128 is fixed for all datasets and electrode configurations.

- **Signal preprocessing**

The raw eight-channel sEMG signal is passed through a third-order Butterworth high-pass filter with a cut-off frequency of 2 Hz and then standardized using per-channel, subject-level z-score normalization, where the mean and standard deviation are estimated from the training repetitions of the corresponding subject only and applied to both the training and test partitions. The normalized signal is segmented into overlapping windows of *T* = 52 samples with a stride of 5 samples, following the windowing convention of [12,15]. Circular padding is applied within the ring topology branch before each convolutional layer along the electrode dimension.

- **Temporal morphology branch**

This branch captures the morphology of motor-unit firing patterns through a multi-scale temporal receptive field. After a convolutional stem that projects the eight-electrode input into 112 feature channels, the signal is processed by four dilated Res2Net-style blocks [36]. Within each block, the input feature map is split into *s* = 4 groups {*u*_1_, *u*_2_, *u*_3_, *u*_4_} along the channel dimension and processed in a cascaded manner:

(1) $$v_{i}=\left\{\begin{array}{ll} u_{i}, & i=1\\ K_{d}(u_{i}), & i=2\\ K_{d}(u_{i}+v_{i-1}), & i>2 \end{array}\right.$$

where *K*_d_ denotes a one-dimensional (1D) convolution of kernel size 7 with dilation *d* ∈ {1, 2, 4, 8} across the four blocks, followed by batch normalization and Gaussian error linear unit (GELU) activation. A channel-wise squeeze-and-excitation (SE) module [37] then recalibrates the channel responses:

(2) $$\beta =\sigma \left(W_{2}\cdot \mathrm{GELU}\left(W_{1}\cdot \mathrm{GAP}(F)\right)\right),\;{\tilde{F}}_{c}=\beta_{c}\cdot F_{c}$$

where GAP denotes global average pooling over time, σ the sigmoid function, and *W*_1_, *W*_2_ the squeeze and excitation projections. The branch outputs a 128-channel feature map, which is adaptively pooled along the time axis to yield *F*_t_.

- **Learnable filter bank branch**

This branch makes the spectral content explicit via a bank of trainable temporal filters, replacing fixed transforms with data-driven, end-to-end-optimized frequency-selective kernels. Each of the *C* electrode channels is convolved independently with *K* = 48 filters, so spectral features are extracted per electrode before any cross-channel mixing:

(3) $$y_{c,k}[t]=\sum_{\tau =0}^{L-1}h_{c,k}[\tau ]x_{c}[t-\tau ]$$

where *L* = 17 is the kernel size and *h*_c,k_ denotes the trainable impulse response of the *k*-th filter on channel *c*. Each filter operates on a single electrode, so spectral information is extracted before any inter-channel mixing. The resulting *C* × *K* = 384 filter responses are linearly combined by a 1 × 1 channel mixing projection:

(4) $$z[t]=W_{\mathrm{mix}}\cdot \mathrm{vec}\left(y[t]\right)$$

where vec(*y*[*t*]) ∈ R^(*CK*)^ stacks all filter responses at time *t*. The output is processed by temporal Res2Net blocks (identical in structure to those of the temporal branch but with independent parameters) and adaptively pooled to yield *F*_f_.

- **Ring topology branch**

The electrodes of an sEMG armband are physically arranged in a closed ring around the forearm, so the first and last channels are direct neighbors even though they are maximally separated along the linear channel index. Most existing models treat the channel axis as linear and therefore break this wrap-around adjacency, discarding a spatial cue that is intrinsic to the sensor geometry. Because the forearm muscles are themselves distributed circumferentially, gesture-related activation patterns can extend across the seam between the first and last electrodes. This branch is designed to preserve this structure. Since each electrode has two physical neighbors and the channel relationship is cyclic rather than linear, the input is padded along the channel axis by circular extension so that channel 1 and channel *C* become neighbors in the padded representation:

(5) $$\bar{x}[c,t]=\left\{\begin{array}{ll} x[C,t], & c=0\\ x[c,t], & 1\leq c\leq C\\ x[1,t], & c=C+1 \end{array}\right.$$

**Figure 2:**
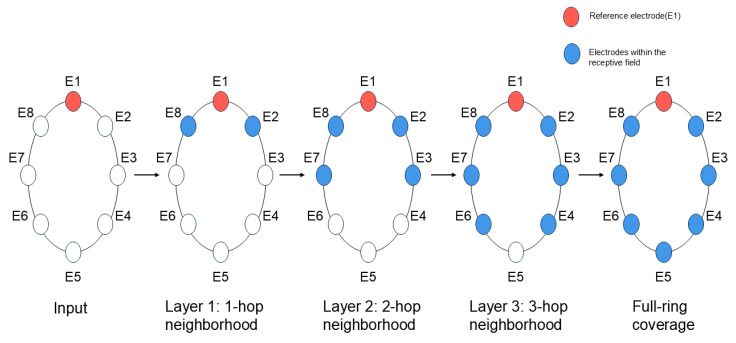
Ring-branch receptive field.

A stack of 2D convolutions with kernels spanning three channels and five temporal samples then aggregates local activation patterns across the ring. This construction is equivalent to a group convolution on the cyclic group *C*_8_ that acts on the electrode index. Because the padding is circular and the kernel weights are shared across positions, a cyclic shift in the electrodes, such as the shift produced when the armband is rotated slightly during donning, induces the same cyclic shift in the branch feature map, so the branch is equivariant to armband rotation rather than merely tolerant of it. The channel extent of the kernel and the depth of the stack are set by the geometry of the ring rather than tuned. A kernel spanning three channels advances the receptive field by one electrode in each direction per layer, which is the smallest possible step on the ring. For *C* = 8 electrodes, the maximum geodesic distance between any two electrodes is ⌊*C*/2⌋ = 4, so four layers is the minimum depth at which every electrode’s representation incorporates the entire ring: three layers reach only seven of the eight electrodes, while additional layers wrap around without covering new electrodes. The stack therefore uses four layers, as illustrated in Figure 2.

At each layer, the ring convolution can be expressed as

(6) $$F_{r}[c,t]=\sum_{\Delta c=-1}^{1}\sum_{\Delta \tau =-2}^{2}W[\Delta c,\Delta \tau ]\bar{x}[c+\Delta c,t+\Delta \tau ]$$

interleaved with batch normalization, GELU activation, and time-only max pooling. The branch consolidates the features into a 128-channel output and adaptively pools it to yield *F*_r_.

- **Adaptive branch fusion**

The relative contribution of each representation may vary across input samples. Therefore, a soft router is used to assign sample-dependent weights to the temporal, filter bank, and ring topology branches. The router considers both the branch feature and two prediction-related cues produced by a lightweight branch-level classification head. For each branch, the classification head converts the branch feature into class logits. The logits are normalized using softmax to obtain the corresponding class probabilities. The maximum softmax probability is used as a confidence cue, while the normalized predictive entropy represents the uncertainty of the branch prediction:

(7) $$p_{b}=\mathrm{softmax}\left(h_{b}(F_{b})\right),\;\;c_{b}=\max_{k}p_{b,k},\;\;e_{b}=-\frac{1}{\log K}\sum_{k=1}^{K}p_{b,k}\log p_{b,k}$$

where $h_{b}(\cdot )$ denotes the branch-level classification head and *K* is the number of gesture classes. The branch-level heads are jointly trained with their corresponding feature encoders. They are supervised using the same ground-truth labels as the final fused classifier. Their auxiliary classification loss is defined as

(8) $$L_{aux}=\frac{1}{3}\sum_{b\in \{t,f,r\}}L_{b}$$

where ${CE}_{LS}$ denotes label-smoothed cross-entropy. The smoothing factor is set to 0.08. The auxiliary loss is incorporated into the overall training objective with a fixed coefficient of $\lambda_{aux}=0.3$. This coefficient controls the strength of the branch-level supervision and is independent of the sample-dependent routing weights.

Before entering the routing module, the confidence and entropy cues are detached from the computational graph. This prevents the routing gradients from directly modifying the branch-level heads. The branch feature and the two detached cues are then passed to the gating multilayer perceptron:

(9) $$s_{b}=g_{\theta }\left(F_{b},c_{b},e_{b}\right),\;b\in \{t,f,r\}$$

The resulting scalar scores are normalized using softmax to obtain the branch weights. The fused representation is calculated as the weighted combination of the three branch features:

(10) $$\alpha_{b}=\frac{\exp(s_{b})}{\sum_{b^{\prime }\in \{t,f,r\}}\exp(s_{b^{\prime }})},\;F=\sum_{b\in \{t,f,r\}}\alpha_{b}F_{b}$$

The fused representation is passed to a fully connected classifier to produce the final gesture class logits. Because the routing scores are computed independently for each input window, the relative contributions of the temporal, filter bank, and ring topology branches can vary across samples. The proposed mechanism therefore provides sample-dependent fusion rather than relying on one fixed set of global branch weights.

The proposed network couples three representation-specific branches with a per-sample fusion router into a single end-to-end model. Each branch contributes a 128-dimensional feature vector that captures one structural property of the signal—the temporal morphology, spectral content, or electrode topology—and the router determines, for every input window, how strongly each property influences the final decision. The network is trained end-to-end with the Adam optimizer with decoupled weight decay (AdamW) (learning rate 6 × 10^−4^, weight decay 2 × 10^−4^) for 120 epochs with a batch size of 256, using a cross-entropy objective with label smoothing. All principal accuracy results reported for the 8-channel subject-dependent NinaPro DB5 setting, the 8-channel subject-adaptive transfer learning setting, the 16-channel dual-armband setting, and MyoArmbandDataset are averaged over three random seeds (42, 3407, and 2026). The ablation experiments use a single fixed seed for computational efficiency.

## 5. Results and Discussion

This work evaluated the proposed method using a standard subject-dependent protocol on the NinaPro DB5 Exercise B benchmark: for 10 subjects, the first one to four repetitions of each gesture were used for training, and the fifth and sixth repetitions were used for testing. Each subject’s model was trained independently, without sharing cross-subject information. Accuracy was calculated over all test windows for each subject, and the resulting subject-level accuracies were then averaged across the ten subjects. The confusion matrix was obtained by pooling the window-level predictions from all subjects and row-normalizing the resulting counts. The main results are averaged over three random seeds (42, 3407, 2026).

### 5.1. Main Results on NinaPro DB5 Exercise B

**Figure 3:**
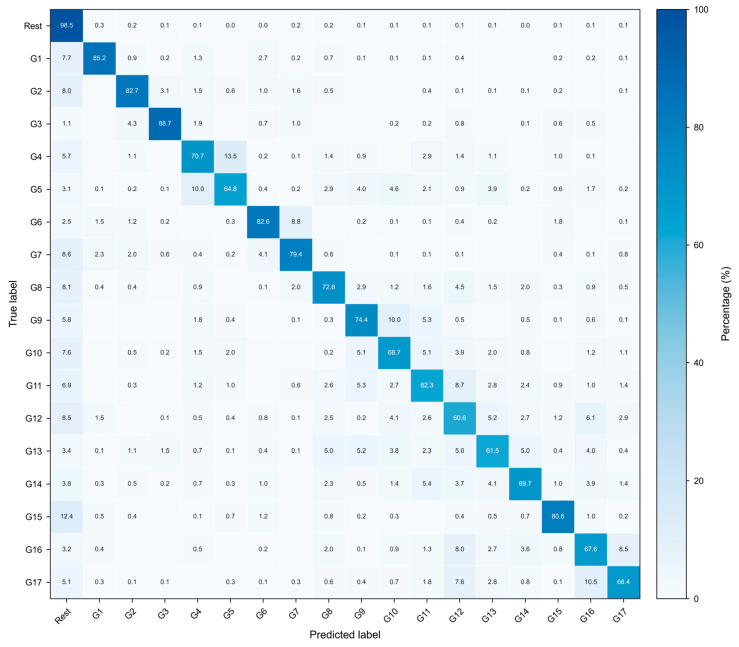
Confusion matrix on NinaPro DB5.

Figure 3 shows the row-normalized confusion matrix on NinaPro DB5 Exercise B, pooled over the ten subjects. The rest class is recognized almost perfectly at 98.5%. The per-class accuracy of the active gestures ranges from 60.6% to 88.7%. The errors are not uniformly distributed, and three patterns can be observed.

First, every active gesture has a small proportion of windows misclassified as the rest class, between 1.1% and 12.4%. Part of this residual error may be associated with onset and offset segments, where muscle activation remains relatively weak. Second, the confusion is concentrated within pairs of kinematically adjacent movements. G4 and G5 are mutually confused at 13.5% and 10.0%. G16 and G17 are mutually confused at 8.5% and 10.5%. G9 is assigned to G10 in 10.0% of the windows. In each pair, the two movements share a similar activation region on the forearm and differ mainly in the direction or the extent of the motion. Third, the wrist movements are more challenging than the hand configurations. The per-class accuracy is between 60.6% and 80.6% for G9 to G17 and between 64.8% and 88.7% for G1 to G8. Among the wrist movement classes, G11–G13 form the weakest subset, with accuracies between 60.6% and 62.3%. These lower accuracies may partly reflect similarities in the corresponding muscle activation patterns, particularly when the movements are differentiated mainly by the relative activation across the electrode ring.

Following the windowing convention of [12,15], the rest class is the most frequent label in Exercise B, since each 5 s movement repetition is followed by a 3 s rest interval. To report the performance independently of this class distribution, we complement the overall accuracy with the macro-averaged F1 score, which weights all eighteen classes equally. RTF-Net attains a macro F1 of 72.4%. The overall accuracy remains the primary metric for consistency with the methods in Table 1, which report the accuracy under the same protocol.

Across three random seeds, the proposed three-branch fusion network achieves mean classification accuracy of 83.99% on NinaPro DB5 Exercise B under the 18-class lower-armband subject-dependent protocol, with a seed-level standard deviation of 0.12 percentage points. Table 1 compares this result with those of representative methods evaluated under the same primary data split without cross-subject pre-training.

On NinaPro DB5 Exercise B (Table 1), RTF-Net achieves accuracy of 83.99 ± 2.65% across the 10 subjects, outperforming the strongest competing method, RCviT [26] (80.23 ± 3.58%), by a mean paired difference of 3.76 ± 1.89 percentage points (95% confidence interval: [2.40, 5.11]). The improvement is significant under both a paired two-tailed *t*-test (t = 6.27, *p* = 0.00015) and a Wilcoxon signed-rank test (*p* = 0.002), with a large effect size (Cohen’s dz = 1.98).

Table 2 reports the same benchmark under a subject-adaptive transfer learning protocol, in which a source model is pre-trained on the remaining subjects and then calibrated on the target subject. Under this protocol, RTF-Net reaches 85.66%. This is 3.61 percentage points above the similarity learning method of Celik and Bayazit [15] and 16.68 percentage points above Raw ConvNet + TL [12]. Relative to the subject-dependent setting of Table 1, the same architecture gains 1.67 percentage points from cross-subject pre-training. The proposed design is therefore not restricted to subject-dependent training, and it also benefits from cross-subject information when such information is available.

Under the subject-adaptive transfer learning protocol (Table 2), RTF-Net achieves 85.66 ± 2.36% accuracy across the 10 subjects. Since Celik and Bayazit [15] report only the aggregate accuracy, a one-sample *t*-test was performed as a benchmark-relative analysis, comparing this subject-level mean against the reported 82.05% (t = 4.83, one-sided *p* = 0.0005; Wilcoxon signed-rank *p* = 0.004; Cohen’s d = 1.53; 95% CI of the mean: [83.97, 87.34]).

Under the subject-dependent protocol in Table 1, RTF-Net achieves 83.99% without cross-subject information. Under the subject-adaptive transfer learning protocol in Table 2, the same architecture reaches 85.66%, corresponding to a gain of 1.67 percentage points. These results indicate that the proposed architecture is effective under subject-dependent training and can further benefit from cross-subject pre-training when such information is available.

### 5.2. Branch Ablation

**Figure 4:**
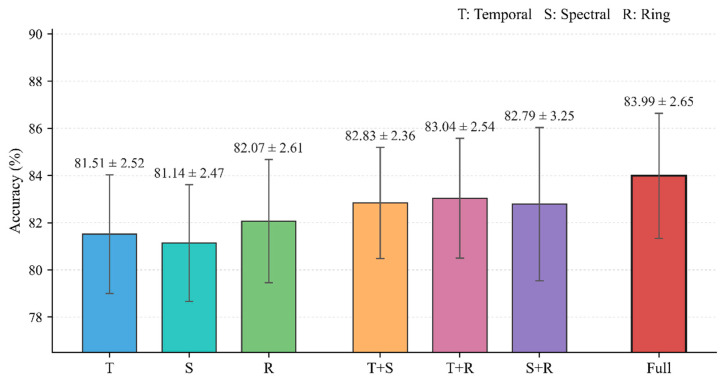
Comparison of ablation experiments on different branches.

As shown in Figure 4, the branch ablation results demonstrate a steady increase in accuracy from the single-branch model to the two-branch combination and then to the complete three-branch model. In the single-branch configuration, the ring topology branch achieved the highest accuracy (82.07 ± 2.61%), outperforming the temporal branch (81.51 ± 2.52%) and the spectral branch (81.14 ± 2.47%). This indicates that the ring electrode topology contains useful spatial discriminative information and enhances the identification of armband-based sEMG signals. The two-branch combination consistently outperformed most single-branch settings, with the Temporal + Ring (T + R) combination showing the best results (83.04 ± 2.54%). However, all two-branch variants underperformed compared to the complete model, which achieved the highest accuracy (83.99 ± 2.65%). These results suggest that the temporal morphology, spectral structure, and ring topology information are complementary.

Although the complete model does not yield the lowest inter-subject standard deviation, its variability remains comparable to that of the ablation variants. The improvement in mean accuracy is therefore not accompanied by a marked increase in cross-subject variability.

**Figure 5:**
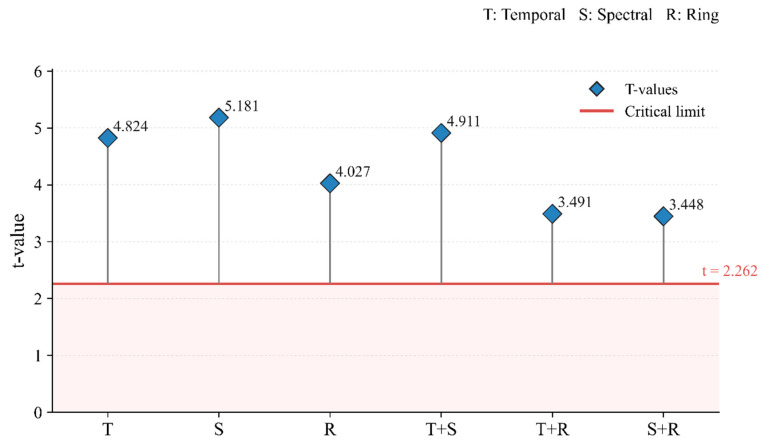
Calculated t-values and critical thresholds for branch ablation.

To further verify whether the observed improvements are statistically significant, Figure 5 shows the calculated t-values from the paired two-tailed *t*-test comparing each ablation configuration with the full model for 10 subjects. All t-values exceeded the critical threshold of 2.262 (*p* < 0.05), ranging from 3.448 to 5.181. These results further support the conclusion that each representation branch contributes to the performance of the complete model. Notably, even the best-performing two-branch configuration significantly underperformed compared to the full model, supporting the conclusion that the representation breadth and the joint integration of all three branches contribute to the final recognition performance.

### 5.3. Fusion Strategy Ablation

As shown in Table 3, the fusion ablation results indicate that simply increasing the number of branches does not guarantee improved accuracy. The mean fusion and static weighting yielded 82.93 ± 2.37% and 82.89 ± 2.45%, respectively, indicating that fixed fusion rules are insufficient to fully utilize the complementary information provided by the three representation branches. Concatenation fusion slightly improved the result to 83.14 ± 2.33%, suggesting that feature-level aggregation is more effective than uniform averaging or globally learned static weights, but the improvement is still limited.

The full router achieves the highest accuracy of 83.99 ± 2.65%, improving on mean fusion by 1.06 percentage points. This result indicates that sample-dependent weighting provides an additional benefit over uniform averaging, globally learned static weights, and feature concatenation. The improvement is moderate but consistent, suggesting that the router is particularly useful for a subset of input windows rather than producing a large global redistribution of branch importance.

It is worth noting that the results for the three-branch static and mean fusion variants are slightly lower than those of the strongest two-branch T + R configuration reported in the branch ablation experiments. This does not diminish the proposed design; rather, it indicates that the breadth of representation and the fusion strategy are coupled factors. Adding a third branch without an effective fusion mechanism may introduce redundant or weakly aligned information, whereas the adaptive router enables the model to exploit complementary cues selectively. Thus, the final improvement comes from the combination of three-branch representation learning and adaptive fusion, not from branch expansion alone.

To isolate the contribution of the confidence-informed routing, we ablate the reliability input to the router while keeping the architecture, the three branches, and the training schedule unchanged. Only the two per-branch reliability scalars fed to the router, namely the maximum confidence and the predictive entropy, are removed. As shown in Table 4, removing both scalars (feature-only gating) reduces the accuracy from 83.99% to 82.95%, corresponding to a drop of 1.04 percentage points. Supplying only one of the two scalars recovers part of this gap, with confidence-only reaching 83.29% and entropy-only reaching 83.32%. This indicates that the two measures are complementary rather than redundant. Although the between-subject standard deviations overlap, the degradation is highly consistent across subjects. The accuracy decreases for all ten subjects when the reliability input is removed, and a paired test confirms that the difference is significant, with a paired *t*-test yielding *p* = 0.0012 and a Wilcoxon signed-rank test yielding *p* = 0.0020. These results support the central design choice of the proposed router, in which conditioning the fusion on each branch’s predictive certainty yields a small but reliable and statistically significant gain.

After examining the contribution of the routing mechanism, this paper further evaluates whether the benefit of the topology branch depends specifically on preserving the physical circular order of the electrodes. Table 5 isolates the contribution of the circular encoding. The architecture, the training schedule, and the parameter count are unchanged, and only the treatment of the channel axis is modified. Replacing circular padding with zero padding reduces the accuracy from 83.99% to 81.82%. The zero-padded variant falls below the two-branch Temporal + Filter (T + S) model at 82.83%, which does not contain the ring branch at all. Adding a spatial branch is therefore not sufficient on its own, and the benefit depends on preserving the wrap-around adjacency between the first and the last electrode. Randomly permuting the channel order before the ring branch reduces the accuracy further to 78.85%, which is 3.98 percentage points below that of T + S. A permutation destroys the physical neighborhood of the electrodes while leaving the signal content unchanged. The ring branch therefore relies on the geometric arrangement of the electrodes and not on the arbitrary mixing of channels.

**Figure 6:**
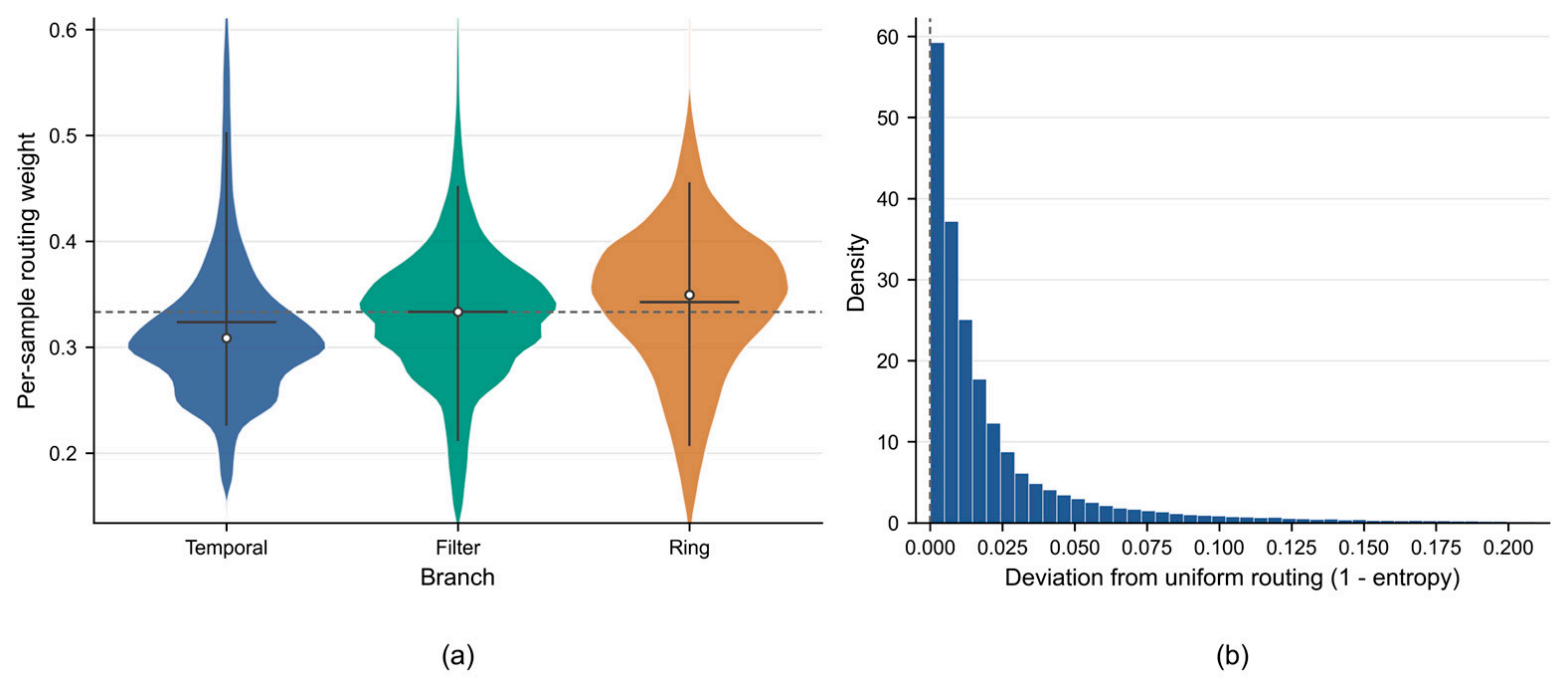
Per-sample routing weights. (**a**) Distribution of the per-sample routing weight of each branch. (**b**) Distribution of the per-sample deviation from uniform routing, defined as one minus the normalized entropy of the weight vector. Dashed lines mark the uniform reference of 1/3 in (**a**) and zero deviation in (**b**).

In addition to evaluating the classification accuracy, we examined whether the router collapses to a single representation or maintains sample-dependent variation. Figure 6 summarizes the distributions of the per-sample routing weights and their deviation from uniform weighting on NinaPro DB5.

Figure 6 characterizes the routing behavior at the sample level. In Figure 6a, the per-sample weights of the three branches are centered close to the uniform reference of 1/3. The ring branch lies slightly above the reference and the temporal branch slightly below. Figure 6b reports the deviation of each sample from uniform routing. The distribution is concentrated near zero and has a long right tail. For most windows, the router stays close to uniform weighting, and only a minority of windows receive a clearly skewed weight vector.

**Figure 7:**
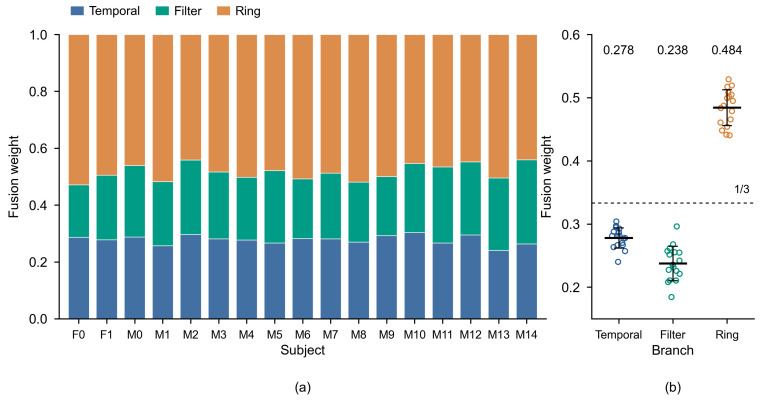
Routing weights of the three branches on MyoArmbandDataset. (**a**) Mean fusion weights of each of the 17 evaluation subjects. (**b**) Distribution of the mean weights of each branch across the 17 subjects, with the group mean and standard deviation. The dashed line marks the uniform reference of 1/3.

This behavior is consistent with the ablation results in Table 4. The router does not reallocate the branch weights globally, and the gain of the full router over feature-only gating is correspondingly moderate at 1.04 percentage points. The gain is nevertheless systematic, since the accuracy decreases for all ten subjects when the reliability cues are removed. The benefit of reliability-aware routing therefore appears to arise primarily from a limited subset of samples for which the branch predictions differ, rather than from a global shift in branch importance. The routing results in Figure 6 were obtained on NinaPro DB5. To examine whether a similar weighting pattern is learned on a second dataset, Figure 7 reports the branch weights obtained on MyoArmbandDataset under the protocol described in Section 3.2.

Figure 7 shows a sharper pattern on MyoArmbandDataset. The ring branch dominates, with a mean weight of 0.484 across the 17 subjects—well above the 1/3 reference—while temporal and filter settle at 0.278 and 0.238; the ordering is stable across subjects. The ring branch is therefore the most heavily weighted representation on both datasets, but the degree of specialization differs: the weights stay close to uniform on the 18-class DB5 protocol and become strongly ring-dominated here. Such dataset-dependent weighting is an aspect that a fixed-fusion baseline could not express.

### 5.4. Calibration Efficiency

A practical challenge in sEMG-based gesture recognition is the need for extensive subject-specific calibration data. The primary NinaPro DB5 protocol used in this study provides four training repetitions per gesture, but reducing this budget is desirable for wearable applications. We therefore evaluated RTF-Net with one to four training repetitions per gesture, keeping repetitions 5–6 for testing and all other settings unchanged.

**Figure 8:**
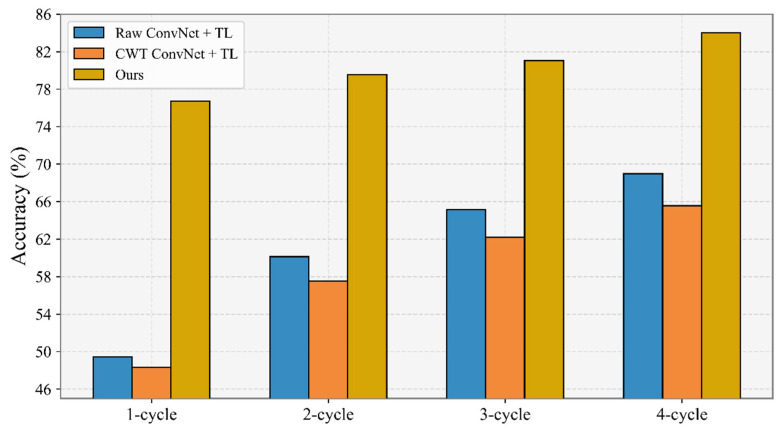
Accuracy as a function of the number of calibration repetitions.

As shown in Figure 8, the accuracy increases monotonically with the number of calibration repetitions, from 76.74% (one repetition) to 79.52%, 81.03%, and 83.99% (four repetitions), with diminishing marginal gains. Reducing the calibration set from four repetitions to one lowers the accuracy by only 7.25 percentage points, indicating that a large part of the recognition performance is retained under a substantially smaller calibration budget. Under each calibration setting, RTF-Net stays above the two transfer learning baselines evaluated at the same number of repetitions, and the margin is largest in the low-data regime. This suggests that the architecture is well suited to short-term calibration scenarios where minimizing the user setup effort is a priority.

Figure 8 also compares RTF-Net with two representative transfer learning baselines, Raw ConvNet + TL and CWT ConvNet + TL [12], evaluated under the same calibration settings, so that all three methods use an identical number of calibration repetitions at each point. Under every setting, RTF-Net stays clearly above both baselines. The advantage is most pronounced in the low-data regime: with a single calibration repetition, RTF-Net reaches 76.74%, whereas both transfer learning baselines remain below 50%, and the gap narrows only gradually as more repetitions are added.

### 5.5. Computational Complexity

As shown in Table 6, the full RTF-Net contains 1.12 M parameters and requires 98.9 million FLOPs per 52-sample window. Despite its three-branch architecture, the model remains computationally feasible for real-time sEMG gesture recognition. On an NVIDIA RTX 3090 GPU, inference runs at 0.051 ms per window (≈19,600 windows/s) in batched mode; even on an Intel Core i9 CPU, a single window is processed in 17.6 ms, well below the 25 ms window interval (stride 5 at 200 Hz). The model therefore meets the real-time requirements on both a GPU and CPU. Notably, the confidence-informed router adds negligible overhead: the feature-only variant has identical parameters and FLOPs and comparable latency (10.84 vs. 10.95 ms), confirming that the accuracy gain from reliability-aware routing comes from information rather than added capacity.

### 5.6. Generalization Across Electrode Configurations and Datasets

This section evaluates generalization along two independent axes: a change in electrode configuration within the same dataset (the 16-channel dual-armband setting on NinaPro DB5) and a change in dataset and protocol (MyoArmbandDataset under subject-adaptive transfer). Together, they test whether the architecture transfers across sensing configurations and across recording conditions without dataset-specific retuning.

Table 7 summarizes the overall performance across electrode configurations and datasets. In the primary NinaPro DB5 Exercise B lower-armband setting, the model achieves 83.99% using only eight sEMG channels under subject-dependent training, where each subject’s model sees only that subject’s own data. Under the subject-adaptive transfer learning protocol of [12,15], in which a source model is pre-trained on the other subjects and then calibrated on the target subject, the accuracy rises to 85.66% on the same eight-channel configuration. We follow the same protocol as in these works to keep the comparison consistent. This protocol is not a strict leave-one-subject-out setting, as is evident from the reported baselines: the transfer learning results of [12] increase steadily as more target calibration data are added, confirming that the target subject’s own data are used during adaptation. The 1.67-point gain over the subject-dependent setting therefore reflects the additional cross-subject information rather than any change in architecture.

Increasing the electrode coverage gives a further improvement. The 16-channel dual-armband configuration reaches 87.17%. In this configuration, circular encoding is applied independently to the two eight-electrode rings before their representations are merged. On MyoArmbandDataset, the model obtains 97.88% under the official pooled protocol. This improves on Raw EMG ConvNet + TL (97.39%) but stays slightly below CWT + TL (98.31%) and MP FT + SVM + kNN (98.77%). This benchmark contains only seven static gestures, and several prior methods already exceed 97% on it. It is therefore close to saturation and offers limited headroom, so these results are best read as a generalization check rather than as primary evidence of superiority.

**Figure 9:**
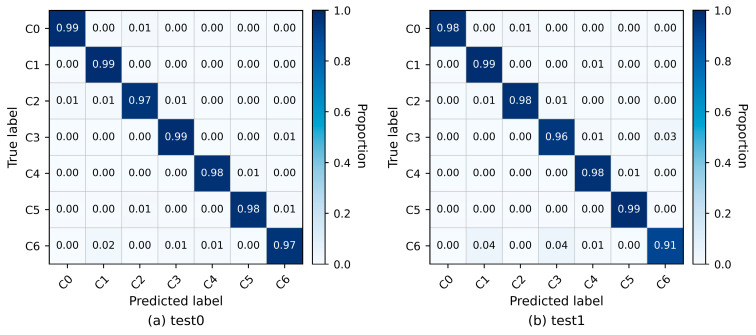
Confusion matrices on MyoArmbandDataset.

Figure 9 presents the row-normalized window-level confusion matrices for the test0 and test1 subsets of MyoArmbandDataset. From a gesture standpoint, C6 (hand open) is the most vulnerable of the seven classes. Hand open is driven by the finger extensors on the dorsal forearm, producing relatively weak and spatially diffuse activation. Its two dominant confusions, C1 (radial deviation) and C3 (ulnar deviation), are wrist movements whose extensor-side activation occupies overlapping regions of the electrode ring, so the low-contrast pattern of hand open is easily mistaken for them. In contrast, hand close (C5) elicits strong, concentrated flexor activation on the volar side and stays well separated, remaining near 0.98–0.99 in both subsets. Hand open is therefore the class that is most sensitive to the electrode shift and skin impedance changes that arise between sessions, consistent with its accuracy holding at 0.97 in test0 but dropping to 0.91 in test1, while the other classes remain stable.

Nevertheless, the main advantage of the proposed method is more evident on the more difficult NinaPro DB5 benchmark, where the gesture space is larger and the classification problem is substantially more challenging. In this setting, the proposed model surpasses the previously reported DB5 results by a clear margin while using the standard lower-armband configuration. Therefore, the MyoArmbandDataset results should be viewed primarily as a generalization check rather than as primary evidence of superiority. In contrast, the DB5 results provide stronger support for the proposed multi-representation fusion design.

## 6. Conclusions and Future Work

This work addresses sEMG-based hand gesture recognition by proposing a three-branch fusion network that explicitly models the signal’s temporal, spectral, and spatial characteristics via dedicated pathways with distinct inductive biases. A temporal morphology branch captures multi-scale motor-unit firing dynamics through dilated residual convolutions; a learnable filter bank branch extracts data-driven frequency-selective responses from each electrode; and a ring topology branch exploits the cyclic spatial arrangement of the electrode armband through circular convolutions. The three branch outputs are integrated by an adaptive per-sample fusion router that learns input-conditional importance weights and produces a unified representation for classification.

On the NinaPro DB5 Exercise B benchmark, RTF-Net attains 83.99% accuracy under subject-dependent training that uses no cross-subject information, and it improves further under transfer learning. The architecture scales with the electrode coverage, generalizes to a second armband dataset without changes to the backbone, and degrades gracefully under reduced calibration budgets.

The ablation studies clarify where this performance arises. Each of the three branches contributes non-redundantly, and the benefit of the ring branch depends on preserving the electrode-ring adjacency rather than on added capacity. Replacing the adaptive router with fixed fusion also lowers the accuracy, and the learned weights vary across samples and datasets in a way that fixed fusion cannot express. Both what to represent and how to combine the representations therefore matter.

Several directions remain open for future investigation. First, although the initial transfer learning experiment shows that RTF-Net can benefit from cross-subject pre-training, a more systematic evaluation of domain adaptation, self-supervised pre-training, and cross-dataset transfer across larger subject populations remains necessary. Second, although the architecture is lightweight (approximately 1.12 M parameters) and adds no inference-time cost beyond the three branches and a small gating MLP, an explicit real-time evaluation on embedded platforms, including latency benchmarking and quantization-aware deployment, would be informative for prosthetic and wearable applications. Third, broader benchmarking across additional sEMG datasets with different sensor counts, sampling rates, and gesture vocabularies would help to characterize the design’s limits. Finally, more fine-grained analyses of routing behavior, including gesture-conditioned, error-conditioned, and temporal analyses, may provide further insight into when and why the three representations are differentially weighted.

## Figures and Tables

**Table 1:** Subject-dependent accuracy on NinaPro DB5.

Method	Accuracy (%)
SVM [6]	64.18
CWT ConvNet, no TL [12]	61.89
Spectrogram ConvNet, no TL [12]	63.60
Raw ConvNet, no TL [12]	66.32
LSTM-CNN [25]	71.40
CWT + EMGNet [24]	67.42
RCviT [26]	80.23
RTF-Net	83.99

Note: Results were taken from the original publications when the reported protocol matched this setting. The SVM [6] and RCviT [26] publications did not report results under this exact protocol and they were reproduced under the same data split; minor differences in preprocessing and implementation may therefore remain for these two methods. The RTF-Net result is averaged over three random seeds (42, 3407, and 2026), with a seed-level standard deviation of 0.12 percentage points.

**Table 2:** Transfer learning accuracy on NinaPro DB5.

Method	Accuracy (%)
Spectrogram ConvNet + TL [12]	65.10
CWT ConvNet + TL [12]	65.57
Raw ConvNet + TL [12]	68.98
CNN-MP + SVM + KNN [15]	82.05
RTF-Net	85.66

Note: Baseline results are quoted from the original publications under the subject-adaptive transfer-learning protocol on NinaPro DB5 Exercise B (see Section 5.6 for protocol details). RTF-Net is averaged over three random seeds.

**Table 3:** Comparison of fusion ablation experiments.

Fusion Strategy	Accuracy (%)	Gain Over Mean	Fusion Type
Static weighting	82.89 ± 2.45	−0.04	Fixed learnable weights
Mean fusion	82.93 ± 2.37	0.00	Uniform averaging
Concatenation fusion	83.14 ± 2.33	+0.21	Feature concatenation
Full router	83.99 ± 2.65	+1.06	Adaptive weighting

**Table 4:** Ablation of confidence-informed routing.

Router Input	Accuracy (%)	Difference From Full Router (pp)
None (feature-only, no reliability)	82.95 ± 3.25	−1.04
Confidence only	83.29 ± 2.84	−0.70
Entropy only	83.32 ± 2.33	−0.67
Full (confidence + entropy)	83.99 ± 2.65	—

**Table 5:** Ring encoding ablation.

Variant	Accuracy (%)	Δ vs. T + S
RTF-Net (circular padding)	83.99 ± 2.65	+1.16
T + S (without the ring branch)	82.83 ± 2.36	—
Ring branch with zero padding	81.82 ± 2.82	−1.01
Ring branch with random channel permutation	78.85 ± 3.74	−3.98

**Table 6:** Model complexity and inference efficiency.

Metric	Value
Parameters	1.12 M
FLOPs (per window)	98.9 M
Latency, GPU batch = 1	10.9 ms
Throughput, GPU batch = 256	≈19,600 windows/s (0.051 ms/window)
Latency, CPU batch = 1	17.6 ms
Throughput, CPU batch = 256	≈1380 windows/s (0.73 ms/window)

**Table 7:** Overall performance across electrode configurations and datasets.

Configuration	Channels	Classes	Subjects	Accuracy (%)
NinaPro DB5 Exercise B, lower armband (primary)	8	18	10	83.99
NinaPro DB5 Exercise B, lower armband, TL	8	18	10	85.66
NinaPro DB5 Exercise B, dual armband	16	18	10	87.17
MyoArmbandDataset (official pooled evaluation)	8	7	17	97.88

## Data Availability

The data upon which this study’s results are based are publicly available at the following websites: MyoArmbandDataset—https://github.com/UlysseCoteAllard/MyoArmbandDataset?tab=readme-ov-file (accessed on 23 July 2026); Ninapro-DB5—https://ninapro.hevs.ch/instructions/DB5.html (accessed on 23 July 2026).

## Abbreviations

The following abbreviations are used in this manuscript:

CNN	Convolutional neural network
CWT	Continuous wavelet transform
GAP	Global average pooling
GELU	Gaussian error linear unit
IMU	Inertial measurement unit
KNN	k-nearest neighbors
LSTM	Long short-term memory
MLP	Multilayer perceptron
RMS	Root mean square
SE	Squeeze-and-excitation
sEMG	Surface electromyography
SVM	Support vector machine
TL	Transfer learning
